# Comparison of Modern Control Methods for Soft Robots

**DOI:** 10.3390/s22239464

**Published:** 2022-12-03

**Authors:** Malte Grube, Jan Christian Wieck, Robert Seifried

**Affiliations:** Institute of Mechanics and Ocean Engineering, Hamburg University of Technology, 21073 Hamburg, Germany

**Keywords:** soft robotics, control, dynamic control, kinematic control, piecewise constant curvature

## Abstract

With the rise in new soft robotic applications, the control requirements increase. Therefore, precise control methods for soft robots are required. However, the dynamic control of soft robots, which is required for fast movements, is still an open topic and will be discussed here. In this contribution, one kinematic and two dynamic control methods for soft robots are examined. Thereby, an LQI controller with gain scheduling, which is new to soft robotic applications, and an MPC controller are presented. The controllers are compared in a simulation regarding their accuracy and robustness. Additionally, the required implementation effort and computational effort is examined. For this purpose, the trajectory tracking control of a simple soft robot is studied for different trajectories. The soft robot is beam-shaped and tendon-actuated. It is modeled using the piecewise constant curvature model, which is one of the most popular modeling techniques in soft robotics. In this paper, it is shown that all three controllers are able to follow the examined trajectories. However, the dynamic controllers show much higher accuracy and robustness than the kinematic controller. Nevertheless, it should be noted that the implementation and computational effort for the dynamic controllers is significantly higher. Therefore, kinematic controllers should be used if movements are slow and small oscillations can be accepted, while dynamic controllers should be used for faster movements with higher accuracy or robustness requirements.

## 1. Introduction

Soft material robots are an emerging and fast-growing field of research with potential applications in various areas. In contrast to conventional robots, which are usually fabricated out of high-stiffness materials such as steel, soft robots are mostly fabricated out of soft materials such as silicone or foam. The material stiffness of soft robots is often in the range of $10^{4}\ldots 10^{9}\;Pa$, which is comparable to the stiffness of biological tissue [1]. Due to the use of soft materials, very large deformations often occur. These are usually used to allow movements of the soft robot without the use of separate rigid joints. Typical applications of soft robots are medical applications and all sorts of human–machine interactions [2].

For the practical application of soft robot, feedback control is often required. As soft robots usually have a very task-specific design, the control methods used are often very specific as well. In this contribution, the focus is mainly on task-space control for trajectory tracking. In Section 1.1, a short overview over existing control approaches is given.

Controller design often requires a model. One of the most popular modeling techniques for soft robots, which will also be used in this paper, is the piecewise constant curvature (PCC) model [3]. This model subdivides a soft robot into sections with constant curvature. The PCC model is described in more detail in Section 2.2.

In this paper, one so-called kinematic control method and two dynamic control methods for tip-position trajectory tracking control of a tendon-actuated soft robot are compared in simulation. For each of the controllers, the achievable accuracy for trajectory tracking, the robustness against parameter uncertainty, the implementation effort and the computation time are examined. The kinematic control method is a model-free closed-loop control approach based on [4,5]. The first examined dynamic control method is a linear quadratic control approach with integral action and gain scheduling, which is novel to soft robotics. The second dynamic control method is a model predictive control approach, which has already been used for the control of different soft robots in the literature. Both dynamic control methods are model-based and closed loop. A PCC model of the soft robot is used for the simulation and for the design of the two model-based controllers.

### 1.1. Related Work

There is a large amount of work related to the control of soft robots. In the following, a short overview is given and summarized in Table 1. The control methods used for soft robots can be divided into two categories: model-based and model-free control. Furthermore, a distinction can be made between kinematic control, where the dynamics of the soft robot are neglected, and dynamic control, where the dynamics of the soft robot are considered [6]. In soft robotics literature, the terms “static controller” and “kinematic controller” are often used interchangeably. Moreover, a distinction can be made between open-loop and closed-loop control [2].

#### 1.1.1. Model-Based Kinematic Control

Model-based kinematic controllers are the most widely used controllers in soft robotics [6]. Most model-based controllers use the piecewise constant curvature (PCC) model [1]. This model is described in Section 2.2 in more detail. However, other models such as the Cosserat rod model are also used [6].

One approach for open-loop model-based kinematic control is to directly invert the forward kinematics, as, e.g., shown by [7]. For the soft robot considered in [7], the deformation depends linearly on the actuation variables. This allows the direct inversion. Since the system is underactuated, the Moore–Penrose pseudoinverse is used.

For more complex soft robots, the relationship between actuation variables and control variables is usually nonlinear and not unique. Therefore, the inverse kinematics cannot be calculated directly. A popular approach here is to use differential inverse kinematics, as, e.g., shown by [8,9,10]. Other approaches to obtain the inverse kinematics are to solve the inversion as an optimization problem [11] or to use an iterative Jacobian transpose approach [12].

#### 1.1.2. Model-Free Kinematic Control

An alternative to model-based kinematic control is model-free kinematic control. Model-free approaches are especially popular for highly nonlinear and nonuniform systems that are difficult to model [6]. In model-free kinematic control, the mapping of actuation variables to control variables is usually learned by a neural network. Model-free kinematic controllers were first proposed in [13]. In that approach, the inverse kinematics of a fully actuated robot are directly learned with a neural network. The training data for the neural network are obtained from the forward kinematics in simulation. In similar approaches, as, e.g., [14], the training data are obtained experimentally from a physical robot.

For redundant soft robots, the relationship between actuation variables and control variables is usually not unique. To achieve a smooth movement, the current configuration of the soft robot has to be considered for the determination of the control variables. This is, e.g., carried out in [4,5,15]. As the current configuration of the soft robot is needed for control, this is a closed-loop control approach. In [16], a combination of open-loop and closed-loop control is used. For the open-loop controller, the kinematics are learned using the multitask Gaussian Process method [35]. The closed-loop control behavior is learned using locally weighted projection regression (LWPR).

Offline training, which is usually very time-consuming, can be fully avoided by using online learning techniques, as, e.g., presented in [17,18]. There, the kinematic Jacobian is determined online by incrementally moving each actuator. However, with this approach, only very low control frequencies can be archived.

#### 1.1.3. Model-Based Dynamic Control

For faster movements and higher accuracy requirements, kinematic control is often not sufficient as the dynamics of the soft robot cannot be neglected anymore. This can be solved by using model-based dynamic control. Compared to kinematic control, dynamic control is much more computationally expensive [6]. Thereby, the PCC model is also one of the most widely used models for model-based dynamic control of soft robots.

One of the first model-based dynamic controllers for soft robots was proposed in [19]. The soft robot is modeled with the PCC model, and the controller is a sliding mode controller. In [20], a closed-loop PD controller based on the same kinematic and dynamic model is presented. Similar control approaches for slightly different soft robots are presented in [21,22,23].

Another very popular control approach that has been successfully applied to soft robots is model predictive control. Model predictive controllers (MPCs) are usually computationally very expensive since, in every control step, the control output is obtained by solving an optimization problem. Therefore, mostly comparatively simple dynamic models of the soft robot are used. In [24], an MPC for an inflatable soft robot is presented. However, in the model used, the soft robot is assumed to be rigid. In [25], a model predictive control approach for soft robots is presented using a linear model with an online update of the Jacobian.

Another approach from classic nonlinear control is adaptive control. Adaptive control is robust to model uncertainties. This is important for in robotics, as, often, model parameters can only be approximated. It is used in [26] for the control of a multi-segment soft robot in 3D. One disadvantage of the formulation of the controller used in [26] is that it can only be used for fully actuated systems. However, soft robots are often underactuated. Finally, in [27], a control law constructed with an energy-shaping approach for a soft robot modeled as a port-Hamiltonian system is presented.

#### 1.1.4. Model-Free Dynamic Control

Model-free dynamic controllers are especially used if no model of the soft robot is available or the available models are too slow for real-time control applications. This is often the case if soft robots are very complex. However, they strongly depend on the availability of training data.

Most model-free dynamic controllers are based on machine learning. In [28,29,30], different reinforcement learning approaches are presented based on a description of the control problem as a Markov decision process. For reinforcement learning, the controllers are usually pretrained in simulation, then the training is continued on the physical robot. A supervised learning approach based on neural networks is presented in [31]. In these approaches, the control output is directly obtained by machine learning.

An alternative approach is to use control concepts known from model-based control but use machine learning techniques to represent the model of the soft robot. This is, e.g., conducted for model predictive controllers in [32,33,34] for different soft robots.

## 2. Modeling

**Figure 1:**
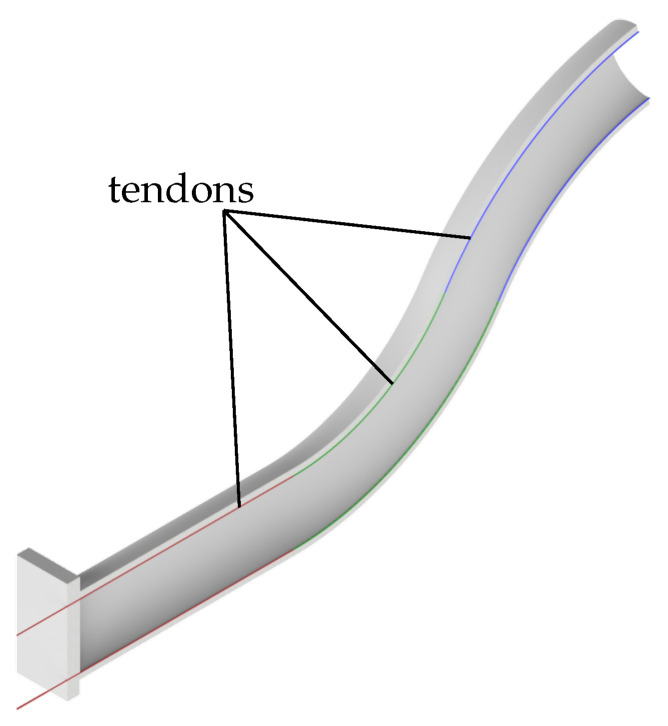
Cut through the soft robot.

In this contribution, a tendon-driven soft robot arm is considered as an application example (see Figure 1). In the following, the used simulation model is presented. First, the design of the model is discussed. Then, the basic piecewise constant curvature model is presented to describe the soft robot. Finally, the inclusion of the cable actuation is discussed.

### 2.1. Simulation Model of the Used Soft Robot

For the comparison of the controllers in this contribution, the control of a simple tendon-actuated soft robot, as shown in Figure 1, is examined in simulation. The model is based on a soft robot of long, slender shape, whose cross-section forms a circular ring with an outer radius of R=50 mm and an inner radius of r=25 mm. The soft robot has a total length of $L_{\mathrm{total}}=500\;mm$ and a Young’s modulus of $E=7.32\;\times \;10^{5}\;Pa$. In this contribution, stiffness proportional damping is assumed with a damping constant of $E\mu =36.6\;\times \;10^{3}\;Nms$. For the actuation, three pairs of tendons run along the top and bottom of the soft robot. The tendons have a distance of $r_{\mathrm{cable}}=25\;mm$ to the neutral fiber. These geometric and material properties are summarized in Table 2. For simplicity, only movements in the xz-plane are considered and modeled. However, this can be extended to 3D in a straight forward way. In the following, only the discretized version of the soft robot is described in more detail.

**Figure 2:**
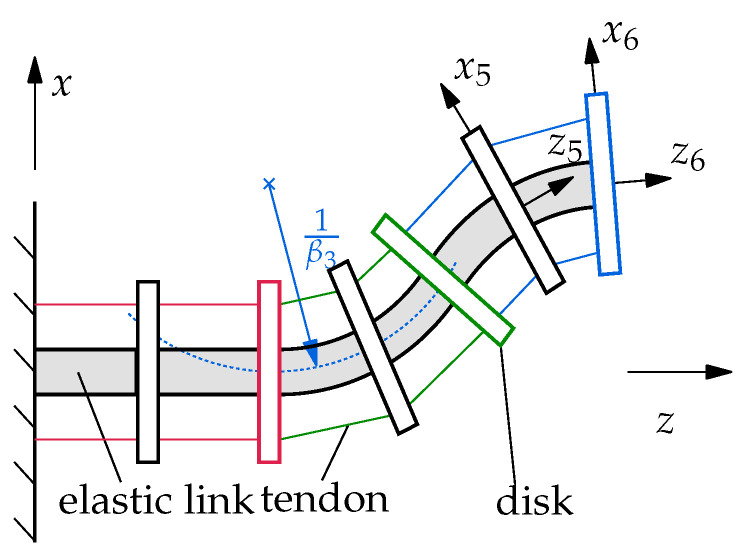
Schematic representation of the 2D system with 6 segments described as the PCC model.

For the discretization, a PCC model with six segments with constant curvature is used. In Section 2.2, the PCC model is described in more detail. The discretized soft robot is shown schematically in Figure 2. For the complete description of the robot configuration, the curvatures $\beta_{i}$ of the individual segments in the xz-plane are sufficient. However, for the full dynamical model, the rate of change of the curvature ${\dot{\beta }}_{i}$ is also required. The model is actuated by three tendon pairs, which run along the outside of the robot. The first tendon pair (indicated in red) ends at the second disk, the second tendon pair (indicated in green) ends at the fourth disk and the third tendon pair (indicated in blue) ends at the sixth disk. The tendon forces of the three tendon pairs are the control variables $u_{1}$, $u_{2}$, $u_{3}$ of the soft robot and can be summarized to the vector of control variables $u\;=\;{\left[u_{1}\;u_{2}\;u_{3}\right]}^{T}$.

Because the soft robot is assumed to be inextensible, only the force difference between the upper and lower tendon of each tendon pair is relevant. As tendons can only transmit pulling forces, it makes sense to assume that for each tendon pair one tendon transmits no force while the other one transmits the required pulling force. Therefore, only one control variable is needed for each tendon pair. A positive sign indicates that the force has to be applied at the upper tendon; a negative sign indicates that the force has to be applied at the lower tendon. In this contribution, it is assumed that all states $\beta_{i}$ and ${\dot{\beta }}_{i}$ are accessible. In practical applications, often, only the curvatures $\beta_{i}$ can be measured directly by sensors integrated into the soft robot, as, e.g., shown in [36,37,38,39]. The rate of change ${\dot{\beta }}_{i}$ of the curvature usually cannot be measured directly due to the lack of suitable sensors. However, as shown in [39], the rate of change ${\dot{\beta }}_{i}$ of the curvature can, e.g., be determined by numerical differentiation with good accuracy.

### 2.2. Piecewise Constant Curvature Model

The piecewise constant curvature (PCC) model is one of the most popular modeling techniques for soft robots. It uses a state-space model to describe the soft robot. The method is presented by [3,40], among others. Compared to other techniques, such as the Cosserat rod theory, it uses fewer modeled degrees of freedom to represent a soft robot. This makes the model less complex than other methods. With a sufficiently fine discretization, it can still reflect the kinematics and dynamics of a soft robot sufficiently well.

The PCC model discretizes a soft robot by a series of *N* segments with constant curvature. In Figure 2, the discretization of a tendon-actuated soft robot with 6 segments is shown. Each of these segments consists of a disk, which is connected to the disk of the previous segment by an elastic link. The inertias and masses of the continuous soft robot are lumped in the disks. The properties of the elastic links are chosen to reflect the elasticity and damping of the real system. According to the model assumption, the links are massless, homogeneous cylinders with constant cross-sections, which have a constant elastic modulus *E* and constant shear modulus. The states of this model are the curvatures $\beta_{i}$ of the sections as well as their derivatives ${\dot{\beta }}_{i}$. This modeling approach is able to represent bending deformations and, in the three-dimensional case, also torsion. Deformations due to shear or strain cannot be represented by this method. However, their influence on the total deformation of the robot is often small compared to bending and torsion and therefore can often be neglected. One advantage of using this model in control is that the curvature of the sections can be directly measured with a suitable sensor on the real robot, while the derivatives of the curvature can be obtained by numerical differentiation [39]. Therefore, in contrast to other methods, there is no need for an advanced state observer.

Following [40], the position $p_{i,\mathrm{local}}$ of disk *i* relative to the position of the previous disk i−1 can be described in a local coordinate frame using the curvatures $\beta_{i}$ as

(1) $$p_{i,\mathrm{local}}\;=\;{\left[\begin{array}{ccc} \frac{1-\cos(\beta_{i}\ell_{i})}{\beta_{i}} & 0 & \frac{\sin(\beta_{i}\ell_{i})}{\beta_{i}} \end{array}\right]}^{T},$$

where $\ell_{i}$ is the length of segment *i*. The global location vector $p_{i}$ of the *i*-th segment results from the location vector of the previous segment and its rotation matrix $R_{i-1}$ to

(2) $$p_{i}=\left\{\begin{array}{cc} p_{i,\mathrm{local}} & i=1\\ p_{i-1}+R_{i-1}p_{i,\mathrm{local}} & i>1 \end{array}\right..$$

The rotation matrix between the local coordinate systems of two neighboring segments is obtained in the two-dimensional case by a simple rotation by the angle $\theta_{i}=\beta_{i}\ell_{i}$ along the *y*-axis. By concatenating these rotations, the rotation matrix from the local coordinate system of the *i*-th disk to the reference system results in

(3) $$R_{i}=\left\{\begin{array}{cc} R_{i,\mathrm{local}} & i=1\\ R_{i-1}R_{i,\mathrm{local}} & i>1 \end{array}\right..$$

The equation of motion also requires the forces $f_{i}$ and torques $\ell_{i}$ acting on each segment. These act at the center of mass of the disk belonging to each segment. The force vector is calculated as

(4) $$f_{i}={\hat{f}}_{i}+f_{i,\mathrm{actuation}},$$

where ${\hat{f}}_{i}$ are external forces and $f_{i,\mathrm{actuation}}$ are forces resulting from the actuation. The torque acting on disk *i* includes the bending torques $\ell_{i,\mathrm{bnd}}$ and $\ell_{i+1,\mathrm{bnd}}$, the damping torques $\ell_{i,\mathrm{dmp}}$ and $\ell_{i+1,\mathrm{dmp}}$, the sum of the external torques ${\hat{\ell }}_{i}$ and the torques due to actuation $\ell_{i,\mathrm{actuation}}$. This results in

(5) $$\ell_{i}=\left\{\begin{array}{cc} \ell_{i,\mathrm{bnd}}-\ell_{i+1,\mathrm{bnd}}+\ell_{i,\mathrm{dmp}}-\ell_{i+1,\mathrm{dmp}}+{\hat{\ell }}_{i}+\ell_{i,\mathrm{actuation}} & i<N\\ \ell_{i,\mathrm{bnd}}+\ell_{i,\mathrm{dmp}}+{\hat{\ell }}_{i}+\ell_{i,\mathrm{actuation}} & i=N \end{array}\right..$$

Under the assumption of linear-elastic material behavior, the bending torques can be calculated as

(6) $$\begin{array}{cc} \ell_{i,\mathrm{bnd}} & =-EJ_{yy}\beta_{i}e_{y}, \end{array}$$

where $J_{\mathrm{yy}}$ is the second moment of area around the *y*-axis, and $e_{y}$ is the unit vector along the *y*-axis. In an analogous way, the damping torques can be determined under the assumption of stiffness-proportional viscous damping to

(7) $$\begin{array}{cc} \ell_{i,\mathrm{dmp}} & =-E\mu J_{yy}{\dot{\beta }}_{i}e_{y}. \end{array}$$

Here, the product Eμ is the damping constant. The derivation of the actuation forces $f_{i,\mathrm{actuation}}$ and actuation torques $\ell_{i,\mathrm{actuation}}$ for a tendon-actuated soft robot is described in Section 2.3.

With the mass $m_{i}$ and inertia tensor $J_{i}$ for all disks, the balance of linear and angular momenta can be established for all disks. Using the direct kinematics and the derived forces $f_{i}$ and torques $\ell_{i}$, from this follows the equation of motion of the form:

(8) $$M\left(\bar{x}\right)\ddot{\bar{x}}=h\left(\bar{x},\dot{\bar{x}},t\right).$$

Here, $M(\bar{x})$ is the mass matrix, and $\bar{x}\;=\;{\left[\begin{array}{cccc} \beta_{1} & \beta_{2} & \cdots & \beta_{N} \end{array}\right]}^{T}$ is the vector of generalized coordinates. With $x\;=\;{\left[\begin{array}{cc} \bar{x} & \dot{\bar{x}} \end{array}\right]}^{T}$, this can also be written as a state-space model:

(9) $$\dot{x}\;=\;\underset{f(t,x)}{\underbrace{\left[\begin{array}{c} \dot{\bar{x}}\\ M^{-1}h \end{array}\right]}}.$$

As an output function,

(10) $$y\;=\;\underset{g(x)}{\underbrace{\left[\begin{array}{ccc} 1 & 0 & 0\\ 0 & 0 & 1 \end{array}\right]\;\cdot \;p_{N}\left(x\right)}}$$

is chosen, which returns the tip position. However, depending on the control problem, different choices are possible. The entire nonlinear differential equation can thus be written as

(11) $$\begin{array}{cc} \dot{x} & =f(t,x),\\ y & =g(x). \end{array}$$

### 2.3. Actuation Forces from Tendon Actuation

Besides pneumatic actuation, tendon actuation, which is used in this contribution, is one of the most popular actuation methods for soft robots. As tendons can only transmit pulling forces, in the planar case, pairs of two tendons have to be used to allow bending the robot in both directions. Thereby, one tendon is placed at the upper side of the robot and the other one at the lower side of the robot. The tendon configuration considered in this work is described in Section 2.1 in more detail.

For the calculation of the actuation forces, friction in the tendon guidance is neglected. Therefore, for each tendon, the tendon force $F_{q}$ is constant over the whole tendon length. Here, the index *q* describes the position of the tendon in the tendon pair, where the upper tendon of the tendon pair has the index 1, and the lower tendon has the index 2. Each tendon pair can be considered separately. Therefore, in the following, only one tendon pair is considered. If, as in this contribution, more than one tendon pair is used, the forces and torques of the different tendon pairs can simply be summed up. A tendon pair can either pass through a disk, end at the disk or not reach this disk. It is assumed that the tendon pair passes the first k−1 disks and ends at disk *k*. Obviously, the largest actuation forces act on the disk where the tendon pair ends, and no actuation forces act on disks that are not reached. Note that actuation forces also act on all disks that the tendon pair only passes.

**Figure 3:**
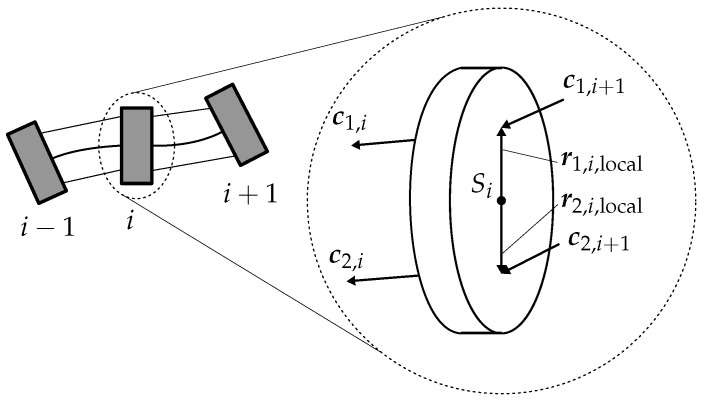
Position of tendon routing points and tendon force vectors on a disk.

For the calculation of the actuation forces, the routing points of the tendons through the disks are of importance. These are shown in Figure 3 for the configuration of tendons used in this contribution. The locations of the routing points in relation to the center of gravity $S_{i}$ of a disk are given by

(12) $$r_{i,1,\mathrm{local}}\;=\;r_{\mathrm{tendon}}{\left[\begin{array}{ccc} 1 & 0 & 0 \end{array}\right]}^{T},$$

(13) $$r_{i,2,\mathrm{local}}\;=\;r_{\mathrm{tendon}}{\left[\begin{array}{ccc} -1 & 0 & 0 \end{array}\right]}^{T}.$$

In global coordinates, these can be written as

(14) $$r_{i,1}=p_{i}+R_{i}r_{i,1,\mathrm{local}},$$

(15) $$r_{i,2}=p_{i}+R_{i}r_{i,2,\mathrm{local}}.$$

From the position of the routing points in global coordinates, the normalized vector $c_{q,i}$ can now be obtained. This vector describes the direction of the tendons—and thus the direction of the tendon forces—from disk *i* to disk i−1. For the *i*-th disk, they are computed by

(16) $$\begin{array}{c} c_{q,i}=\frac{r_{i-1,q}-r_{i,q}}{∥r_{i-1,q}-r_{i,q}∥}. \end{array}$$

The forces $f_{\mathrm{tendon},i}$ acting on disk *i* can now be calculated as

(17) $$\begin{array}{c} f_{\mathrm{tendon},i}=\left\{\begin{array}{cc} \sum_{q=1}^{2}c_{q,i}F_{q}-c_{q,i+1}F_{q} & i<k\\ \sum_{q=1}^{2}c_{q,i}F_{q} & i=k\\ 0 & \mathrm{otherwise} \end{array}\right.. \end{array}$$

In an analogous way, the resulting torques $\ell_{\mathrm{tendon},i}$

(18) $$\begin{array}{cc} \ell_{\mathrm{tendon},i} & =\left\{\begin{array}{cc} \sum_{q=1}^{2}\left(\left(R_{i}\;\cdot \;r_{i,q,\mathrm{local}}\right)\times \left(c_{q,i}F_{q}-c_{q,i+1}F_{q}\right)\right) & i<k\\ \sum_{q=1}^{2}\left(\left(R_{i}\;\cdot \;r_{i,q,\mathrm{local}}\right)\times \left(-c_{q,i}F_{q}\right)\right) & i=k\\ 0 & \mathrm{otherwise} \end{array}\right. \end{array}$$

can also be obtained.

## 3. Control Methods

In the following, the three examined control methods are briefly presented.

### 3.1. Kinematic Control

The first considered controller uses a model-free, kinematic approach based on [4,5]. Here, a shallow neural network is used to learn the global inverse kinematics of a soft robot. This can then be used to determine the necessary control values for a desired configuration of the robot. The dynamics of the system are neglected.

In general, the direct kinematics of the soft robot can be denoted as

(19) y=h(u)

with the vector of the control variables u and the resulting steady-state tip position y. To determine the inverse kinematics, the direct kinematics from Equation (19) have to be inverted. Since the considered soft robot is kinematically redundant, there exists no unique global solution for this; the function h is surjective. However, the inverse differential kinematics

(20) δy=J(u)δu

can be used to obtain a locally unique solution [41]. Here, J(u) is the Jacobian-matrix of h(u) with respect to the control variables u. The variations of the tip position y and the control variables u are denoted δy and δu, respectively. Following [5], Equation (20) can be discretized such that

(21) $$y_{j+1}-y_{j}=J\left(u_{j}\right)\left(u_{j+1}-u_{j}\right).$$

Here, $u_{j+1}$ is the vector of control variables that reaches the positions $y_{j+1}$ starting from the current position $y_{j}$ with the corresponding control variables $u_{j}$. In general, Equation (21) only holds if the difference between the control variables $u_{j}$ and $u_{j+1}$ is infinitesimally small. In practice, [41] shows that the equation also provides usable approximate solutions for larger distances.

**Figure 4:**
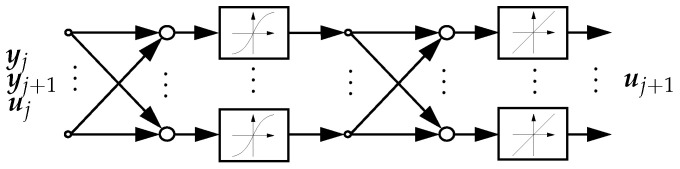
Structure of the neural network of the kinematic controller.

For the kinematic control, Equation (21) must be solved for the control variable vector $u_{j+1}$. This results in

(22) $$u_{j+1}=J{\left(u_{j}\right)}^{†}\left(y_{j+1}-y_{j}+J\left(u_{j}\right)u_{j}\right)=z\left(y_{j},y_{j+1},u_{j}\right)$$

with $J{\left(u_{j}\right)}^{†}$ as the Moore–Penrose pseudoinverse of the Jacobian matrix J. The mapping function of $y_{j}$, $y_{j+1}$ and $u_{j}$ to $u_{j+1}$ is denoted as z in the following. It represents a locally valid inversion of the kinematics of the soft robot. For the soft robot model considered in this contribution, the function of the local inverse kinematics z cannot be determined analytically. Therefore, in the following, it is approximated with a neural network with one hidden layer. The structure of the neural network is shown in Figure 4. The input layer has seven neurons. The hidden layer has 30 neurons and uses the tangent hyperbolicus as activation function. The output layer has three neurons and uses a linear activation function. The training is performed with Bayesian regularization.

**Figure 5:**
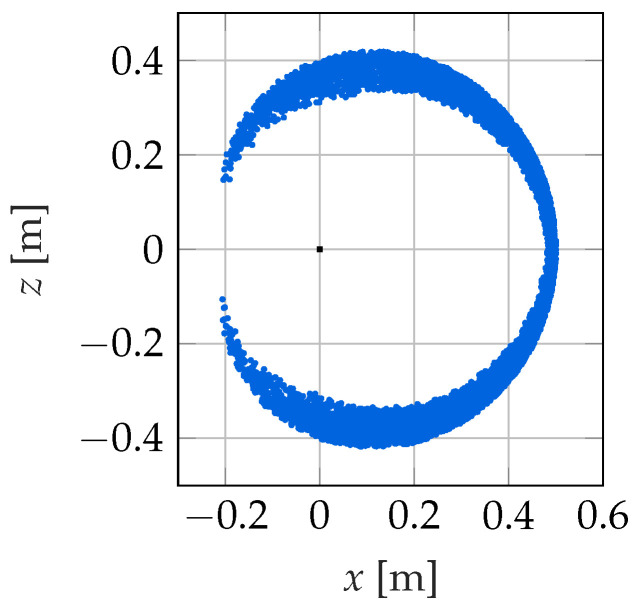
Empirically determined workspace of the kinematic controller for $N_{\max}=20\;N$.

For the training of the neural network, a sufficient number of value pairs of the mapping $\left(y_{j},y_{j+1},u_{j}\right)\to u_{j+1}$ are required. To determine the required training data, the simulation model described in Section 2.1 is used. For the generation of one pair of values, the vector of control variables $u_{j}$ and the vector of varied control variables $u_{j+1}$ are chosen randomly such that $u_{j}\in \left[-N_{\max},N_{\max}\right]$ with $N_{\max}=20\;N$ and

(23) $$|u_{j}-u_{j+1}|<\varepsilon N_{\max}$$

with ε=5%. Then, the robot is excited with the vector of control variables $u_{j}$, and the resulting initial position $y_{j}$ is determined after the transient processes have decayed. Subsequently, the control variables are changed to the new vector of control variables $u_{j+1}$, which results in the corresponding position $y_{j+1}$. In this way, a complete pair of values of the mapping $\left(y_{j},y_{j+1},u_{j}\right)\to u_{j+1}$ is determined. This procedure is repeated 10,000 times to collect the training data for the neural network. This takes about 5min on a PC with an “Intel Core i7 - 6700K” processor. The resulting workspace of the soft robot is shown in Figure 5 and is reasonable and realistic for this type of robot.

**Figure 6:**
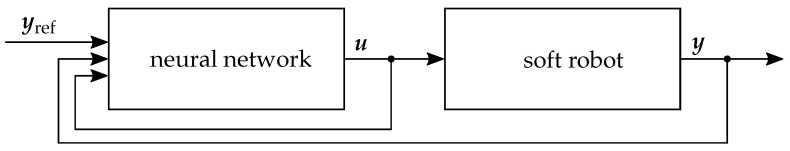
Block diagram of the closed control loop with the neural network as controller.

The trained neural network can now be used as a controller for the soft robot, as shown in Figure 6. The inputs of the neural network are the current tip position $y_{j}$, the current control variables $u_{j}$ and the desired tip position $y_{\mathrm{ref}}$. The outputs of the neural network are the control variables $u_{j+1}$ that are required to archive the desired tip position. The controller runs with a sample frequency of $f_{\mathrm{controller}}=\;5\;Hz$. The reason for this comparatively low frequency is the kinematic behavior of the controller neglecting the dynamics of the soft robot. With each change in the control output u, the dynamics of the system are excited; however, the kinematic controller assumes that the steady-state position is reached instantaneously. With the low sample frequency, the transients can at least partly decay before the measurements for the next control step are taken, which improves the performance and the stability of the controller. On the other hand, if the sample frequency of the controller is chosen even lower, the robot can only move very slowly.

### 3.2. Linear Quadratic Control with Gain Scheduling

The second controller considered is a linear quadratic controller with integral action (LQI controller) and gain scheduling. This is an approach from optimal control that has not been applied to soft robotics so far. In general, for the control of linear time-invariant systems of the form

(24) $$\begin{array}{cc} \dot{x} & =Ax+Bu,\\ y & =Cx \end{array}$$

LQI controllers can be used [42]. Here, x is the state vector, u the input vector and y the output vector. The dynamic behavior of the linear system and the relationship between the input vector u, the states x and the output y are described by the matrices A, B and C. As LQI controllers are extensively discussed in the literature, e.g., [43,44], they are not described in further detail here.

The controller considered so far requires a linear model of the system to be controlled for the design. However, the soft robot used in this contribution has nonlinear behavior, which can be described as

(25) $$\begin{array}{cc} \dot{x} & =f(x,u),\\ y & =g(x). \end{array}$$

**Figure 7:**
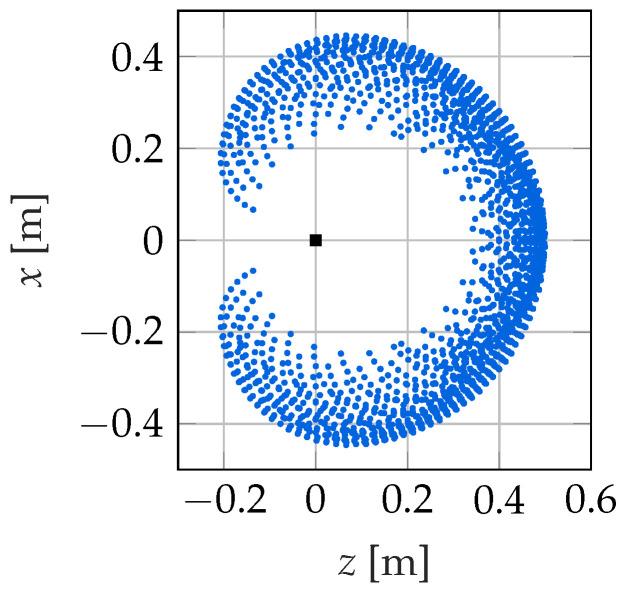
Tip position and spanned workspace of the LQI controller.

In order to derive an LQI controller for such a nonlinear system, it has to be linearized [45]. In this paper, the nonlinear system is therefore linearized around 2288 operation points OP, which are regularly distributed in the workspace. The operation points are chosen such that the velocities of the soft robot are zero in these points. They are shown in Figure 7. Each operation point consists of a state vector $x_{\mathrm{OP}}$, a control variable vector $u_{\mathrm{OP}}$ and the corresponding system output $y_{\mathrm{OP}}$. However, as long as no external forces are considered, as in this contribution, three parameters are sufficient to describe an operation point because the steady-state configuration of the soft robot then only depends on the three tendon forces $u_{1}\ldots u_{3}$. Here, the curvature of the first, third and fifth segment, $\beta_{1}$, $\beta_{3}$ and $\beta_{5}$, is chosen. These can be collected in the scheduling vector $\sigma ={\left[\beta_{1}\;\beta_{3}\;\beta_{5}\right]}^{T}$.

Given the state function f(x,u), the output function g(x) and the operating point, the matrices of the state space representation of the linearized system can be determined. These are calculated, as shown by [45], among others, as

(26) $$\begin{array}{cc} \tilde{A} & ={\left.\frac{\partial f}{\partial x}\right|}_{x_{\mathrm{OP}},u_{\mathrm{OP}}}, \end{array}$$

(27) $$\begin{array}{cc} \tilde{B} & ={\left.\frac{\partial f}{\partial u}\right|}_{x_{\mathrm{OP}},u_{\mathrm{OP}}}, \end{array}$$

(28) $$\begin{array}{cc} \tilde{C} & ={\left.\frac{\partial g}{\partial x}\right|}_{x_{\mathrm{OP}},u_{\mathrm{OP}}}. \end{array}$$

This results in the linearized system

(29) $$\begin{array}{cc} \tilde{\dot{x}} & =\tilde{A}\tilde{x}+\tilde{B}\tilde{u},\\ \tilde{y} & =\tilde{C}\tilde{x} \end{array}$$

with $\tilde{x}=x-x_{\mathrm{OP}}$, $\tilde{y}=y-y_{\mathrm{OP}}$ and $\tilde{u}=u-u_{\mathrm{OP}}$.

**Figure 8:**
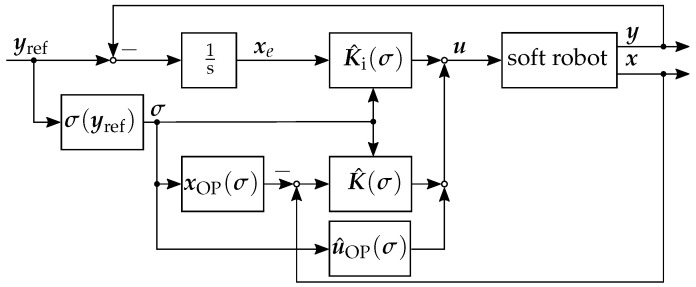
Block diagram of the closed control loop with the LQI controller with gain scheduling.

For each of the linearized systems in the operation points, a separate LQI controller is now designed. For the control of the soft robot, in each time step, a trilinear interpolation is performed between the eight controllers spanning the cuboid in the three-dimensional parameter space in which the current scheduling parameter σ lies. In Figure 8, the block diagram of the LQI controller with gain scheduling is shown. The control law results in

(30) $$u\left(\sigma \right)=K\left(\sigma \right)\left(x-x_{\mathrm{OP}}\left(\sigma \right)\right)+K_{i}\left(\sigma \right)\int \left(y-y_{\mathrm{ref}}\right)\mathrm{dt}+u_{\mathrm{OP}}\left(\sigma \right).$$

Here, the gain matrix of the controller is subdivided into the gain $K_{i}$ of the integrated control error $x_{e}=\int \left(y-y_{\mathrm{ref}}\right)$ for the integral behavior and the gain matrix K for the proportional and derivative behavior of the controller.

### 3.3. Model Predictive Control

**Figure 9:**
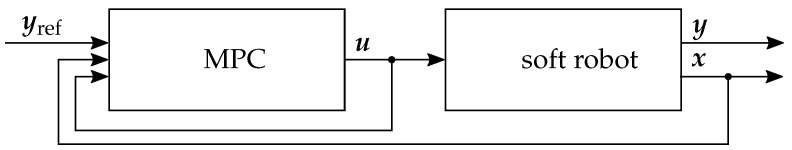
Block diagram of the closed control loop with the model predictive controller.

The third control method considered is a model predictive controller (MPC). The block diagram of this controller is shown in Figure 9. This discrete, model-based method is, e.g., used by [25] for the control of a soft robot. The MPC solves an optimization problem in each timestep of the control in order to determine the best possible control output *u*. For this purpose, in every timestep, the controller optimizes the control output for the next *c* timesteps (control horizon) such that the control error and control effort over the next *p* timesteps (prediction horizon) is minimized. The optimization problem is described by

(31) $$\begin{array}{c} \min_{p\;\in \;P}J\left(x\left(k\right),u\left(p\right)\right)\;P=\left\{z\in R^{m\;\cdot \;c}\right\} \end{array}$$

with the cost function

(32) $$\begin{array}{cc} J(x(k),u) & =\sum_{i=1}^{p}\left(e{\left(k+i\right)}^{T}Qe\left(k+i\right)+u{\left(k+i\right)}^{T}Ru\left(k+i\right)+\Delta u{\left(k+i\right)}^{T}R_{\Delta }\Delta u\left(k+i\right)\right) \end{array}$$

where

(33) $$\begin{array}{cc} e(n) & =y_{\mathrm{ref}}\left(n\right)-y\left(n\right), \end{array}$$

(34) $$\begin{array}{cc} \Delta u(n) & =u(n)-u(n-1). \end{array}$$

Here, Q, R and $R_{\Delta }$ are weighting matrices to specify the influence of the different cost terms. In total, m·c variables have to be computed in an optimization, where *m* is the number of control variables. The parameters for the optimization are obtained by combining the control variables of the individual time points in the control horizon in the vector

(35) $$p={\left[\begin{array}{cccc} u_{k+1}^{T} & u_{k+2}^{T} & \cdots & u_{k+c}^{T} \end{array}\right]}^{T}.$$

Here, $u_{n}$ is the control variable that applies to the time step with index *n* in the control horizon. To solve the optimization problem, in every timestep, the nonlinear soft robot model integrated into the controller has to be evaluated several times.

In this paper, a sample time of $T_{s}=0.1\;s$, a prediction horizon of p=5 steps and a control horizon of c=3 steps are chosen. Usually, better control results can be archived with a shorter sample time and longer prediction and control horizons. However, the computational costs also increase because the optimization problem is harder to solve.

The controller is implemented in Simulink with the *Model Predictive Control* Toolbox. The internal model is integrated with the explicit Euler method with stepsize $T_{s}$. The optimization problem is solved with the Matlab function *fmincon*. It uses the SQP method for the minimization [46]. With the chosen implementation of the MPC in Simulink, a time delay of one control step (0.1 s) is induced. However, with a different implementation of the MPC, this could be avoided.

## 4. Results

In the following, for all three controllers, the trajectory tracking results as well as the results of the examination of the robustness against parameter uncertainty are presented. Thereby, the control error of the tip position is calculated for an arbitrary time *t* as

(36) $$e\left(t\right)=∥y\left(t\right)-y_{\mathrm{ref}}{\left(t\right)∥}_{2}.$$

### 4.1. Test Trajectories

**Figure 10:**
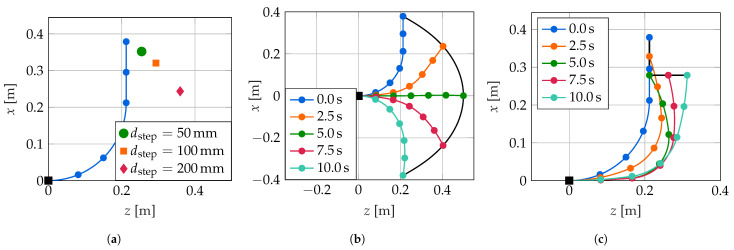
Examined trajectories. (**a**) Step trajectory. (**b**) Swing trajectory. (**c**) L-shape trajectory.

For the examination of the performance of the three controllers, three different types of trajectories are considered: A step change in the tip position, a swing trajectory passing through nearly the whole workspace and an L-shaped trajectory. These are shown in Figure 10. For all trajectories, only the tip position is defined; the controllers are free to choose any configuration of the soft robot that reaches that tip position. The configurations shown in Figure 10 are the configurations archived with the LQI controller. Note that the step trajectory is not continuous, and all three trajectories are not differentiable. Therefore, the controllers cannot follow the trajectories exactly.

The step trajectory is the most basic trajectory of the three considered trajectories and is especially popular in linear control but is also widely used for nonlinear systems. Here, three different step widths of $d_{\mathrm{step}}=50\;mm$, $d_{\mathrm{step}}=100\;mm$ and $d_{\mathrm{step}}=200\;mm$ are examined. The swing trajectory crossing through nearly the whole workspace is the most natural movement of the soft robot. Starting at rest from an initial position, the soft robot has to reach its final position within 10 s with constant velocity and come to a rest there. The trajectory is not differentiable at its beginning and at its end due to the desired constant velocity. The L-shape trajectory consists of two axis-parallel movements with constant velocity that take 5 s each. Again, the soft robot has to come to a rest at the final position. This movement is less natural and can only be archived with larger curvatures.

### 4.2. Step Trajectory

**Figure 11:**
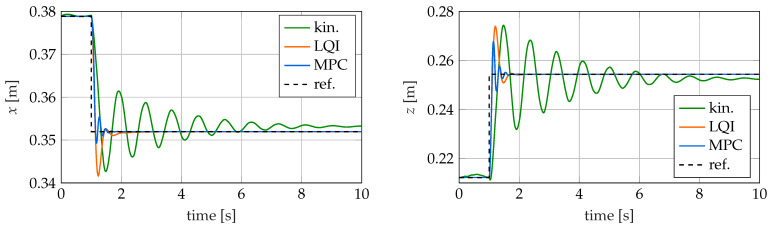
Reference trajectory as well as the tip position for different controllers with $d_{\mathrm{step}}=50\;mm$ over time.

As the first control scenario, the step change in the tip position is examined, starting with the stepsize $d_{\mathrm{step}}=50\;mm$. In Figure 11, the tip position is plotted over time in x− and z−direction for all three controllers together with the desired trajectory. Characteristic quantities for the step responses are the rise time, the settling time, the overshoot and the remaining control deviation. These are listed in Table 3. Here, the rise time is the time the response takes to rise from 10% to 90% of the way from the initial value to the steady-state value. For the settling time, a band of 2% around the final rest position is considered.

It can be seen that all control methods considered are capable of bringing the tip of the soft robot to the desired reference position, but the quality of control partly differs clearly between the methods. It is particularly noticeable that the kinematic controller has more than twice the rise time compared to the two dynamic controllers. At the same time, the settling time of the kinematic controller is also about one order of magnitude larger. This can be explained by the fact that the kinematic controller does not consider the dynamic behavior of the soft robot. Therefore, it cannot actively damp the excited oscillations of the robot. This leads to a significantly longer settling time and reduces the maximum useful speed of the robot. The LQI and the MPC controller achieve a comparably low rise time. However, with 0.49 s, the settling time of the model predictive controller is about 45% lower than the settling time of the LQI controller. This can be explained by the predictive control behavior of the model predictive controller. This also explains the about 15% lower overshoot of the model predictive controller compared to the other two controllers considered.

Both the LQI and MPC controllers exhibit a negligible steady-state error. For the LQI controller, this can be explained by the integral control behavior. The model predictive controller achieves a negligible control error because its internal prediction model matches the soft robot model used exactly. Therefore, the necessary control variables can be determined exactly from the optimization problem. The kinematic controller has a steady-state error of 4.85%. This can be explained by the used neural network, which only represents an approximation of the inverse kinematics.

For larger steps of $d_{\mathrm{step}}=100\;mm$ and $d_{\mathrm{step}}=200\;mm$, the behavior of the controllers is qualitatively comparable. The characteristic quantities for the step responses with $d_{\mathrm{step}}=200\;mm$ are listed in Table 4. Only the settling time of all controllers nearly doubles or triples. This is especially noticeable for the kinematic controller since it has a very long settling time anyway. Therefore, large step sizes should be avoided when using the kinematic controller.

The calculation time of all three controllers is sufficient for real-time applications. The kinematic controller requires 5.4 μs per evaluation, which is much lower than the sample time $T_{S}=200\;ms$ of the controller. Note that, as described in Section 3.1, even though the computation time is very low, the sample time should not be chosen much lower to keep the controller stable. With 11.2 μs per evaluation, the LQI controller is slightly slower. The model predictive controller takes 49.7 ms for the calculation of one control step. As expected, it has the longest computation time per evaluation of the three considered controllers. Nevertheless, the model predictive controller with a used sampling time of $T_{s}=\;0.1\;s$ is also real-time capable on the used hardware and with the chosen prediction and control horizons. The calculation times and sample times of the controllers are listed in Table 5. For the other trajectories, comparable computation times are archived. These will therefore not be discussed further in the following.

### 4.3. Swing Trajectory

**Figure 12:**
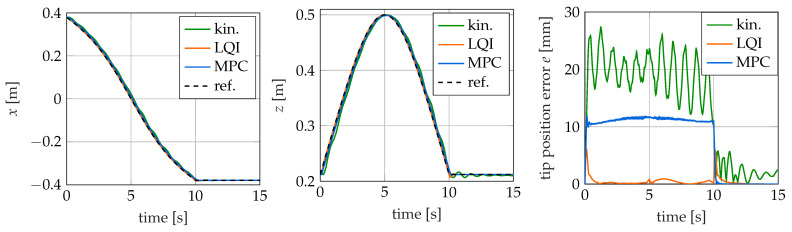
Reference trajectory as well as the tip position for different controllers for the swing trajectory over time.

The time courses of the trajectories in *x*- and *y*-directions, as well as the position error *e*, are shown in Figure 12 for the three examined controllers. It can be seen that all the examined controllers are in principle able to follow the given trajectory. However, the control error *e* of the controllers differs significantly.

The largest control error of all controllers is observed for the kinematic controller. The maximum error is 27.3 mm (5.46% related to the total length of the soft robot), which is more than twice the peak error of the model predictive controller and four times that of the LQI controller. From the plots of the tip position over time, it is evident that the kinematic controller oscillates around the nominal trajectory. This can be mainly explained by the controller neglecting the dynamic characteristics of the soft robot. In addition, there are inaccuracies in the prediction of the required values for the control variables by the neural network. The kinematic controller also takes the longest time after the completion of the swing motion to reach the final position. Within the additional time of 5 s considered after the completion of the motion, it does not succeed in reaching the target position completely. Considering longer simulation times, the oscillation decays due to the internal damping of the soft robot, and a permanent control error of 1.7 mm (0.34%) remains.

The LQI controller shows the smallest control error of the examined controllers with a maximum error of 6.13 mm (1.23%) shortly after the motion starts. A second significant error occurs at time t = 10 s when the given swing motion ends and the tip remains in the final position. The cause of both errors is that the swing trajectory, as described in Section 4.1, is not continuously differentiable at these points. Therefore, here, the transient behavior of the system and the overshoot behavior of the controller can be observed. For the rest of the motion, the error is smaller than 1 mm (0.2%).

The model predictive controller has a maximum control error of 11.77 mm (2.35%). The errors occurring during the movement are nearly constant at that level. They vary by only about 1 mm during the trajectory. This can be seen in Figure 12 where the tip trajectory as well as the control error are plotted over time. The reason for the control error is mainly the used implementation of the MPC, which, as described in Section 3.3, induces a small time lag. The lag also shows in the tip trajectories as a nearly constant offset during the motion. This cause is also supported by the fact that the control deviation drops to nearly zero as soon as the swing movement ends and the reference signal is constant.

**Figure 13:**
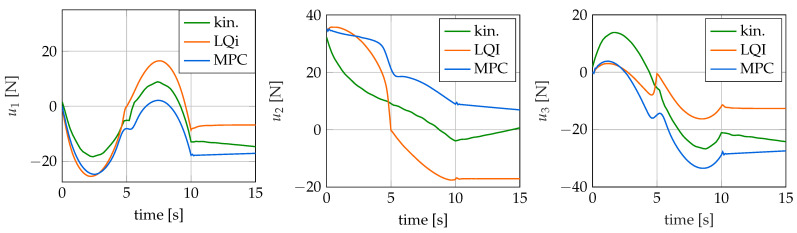
Actuation forces for the swing trajectory over time.

In addition to the time courses for the tip position and the control error, the values for the control variables applied by the controllers are also of interest. These are shown for the different controllers in Figure 13 over time. Note that a positive force is an actuation of the upper tendon and a negative force is an actuation of the lower tendon. It is noticeable that all controllers calculate different values for the control variables. The basic form of the curves and the total control effort are similar, but the actual values differ by up to 29 N. Large differences are particularly noticeable for the LQI and the model predictive controller. This can be seen especially well at the end of the simulation time where both the LQRI and the MPC have a negligible control error. Furthermore, both controllers calculate different control forces. However, it follows from the different values for the control variables that the curvatures of the soft robot archived by the controllers are also different. This can be explained by the controllers finding different robot configurations that lead to the same tip position. This is also true, to a certain extent, for the kinematic controller, although it does not reach the target position completely. The configuration determined by it is also different from those of the other two controllers. The differences in the actuation forces calculated by the three controllers are only possible because the soft robot model is a kinematically redundant system. This means that there are different configurations and hence values for the control variables that lead to the same tip position.

### 4.4. L-Shape Trajectory

**Figure 14:**
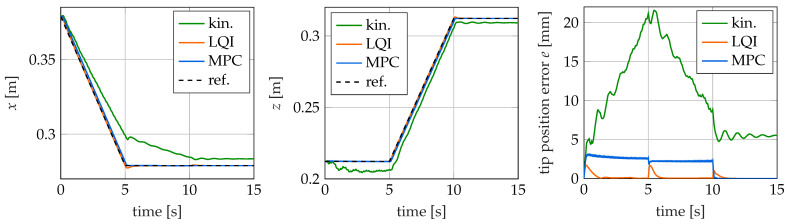
Reference trajectory as well as the tip position for different controllers for the L-shape trajectory over time.

**Figure 15:**
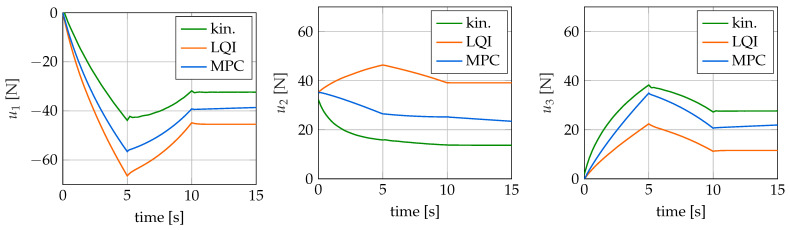
Actuation forces for the L-shape trajectory over time.

The results for the control of the L trajectory are shown in Figure 14 and Figure 15 and largely match those already observed for the swing trajectory in Section 4.3. In particular, they turn out even better for the dynamic controllers.

For the kinematic controller, the control error is again significantly larger than for the other two controllers. Thus, with a maximum value of 21.57 mm (4.31%), this is at the same level as for the swing motion. From Figure 14, it is clear that the controller has particular difficulties in reaching the final position along the *x*-axis. The deviation at time t = 5 s for this component is 20.37 mm. A possible reason for this very large deviation, also compared to the swing motion, is the considered workspace. For the design of the kinematic controller in Section 3.1, maximum actuating forces of ±20 N are considered. The controller does not have any data from the design about the relationship between the control variables and the tip position outside this area and therefore has to extrapolate there. However, to follow the trajectory, actuation forces of ≈±40 N are required. The control variables calculated by the neural network therefore differ from those actually required. Only at the end of the movement, when the required tip position is again in the workspace of the kinematic controller, does the occurring control error decrease again. If the time simulation is continued for a longer simulation duration, a permanent control error of 5.47 mm (1.09%) is obtained, which is significantly higher than the permanent error for the swing motion.

The maximum control error of the LQI controller with 1.71 mm (0.34%) is below the value determined for the swing motion. It is well recognizable that the position deviation always increases abruptly at the times when the trajectory is not continuously differentiable. The smaller deviation, compared to the swing motion, is due to the fact that the motion is slower because of the shorter trajectory. Thus, the steps in the velocity course of the trajectory are smaller at the points where it is not continuously differentiable. It is therefore easier for the controller to follow the trajectory and to compensate for the error that occurs. Apart from the three sudden increases and the following decay, the control error of the LQI controller is below 0.2 mm (0.4%).

With a maximum error of 3.11 mm (0.62%), the model-predictive controller also has a significantly lower control error compared to the swing trajectory. As with the latter, the deviation is at a roughly constant level during the motion. The lower occurring control error is, just as in the case of the LQI controller, due to the slower speed of the movement. As the control error is mainly caused by the lag, as explained in Section 4.3, it is nearly proportional to the velocity. This is again supported by the fact that the deviation quickly approaches zero as soon as the reference trajectory has reached the end position.

Finally, the control variables calculated by the controllers for the executed movement are considered. These are shown in Figure 15 for the individual controllers over the simulation time. Once again, it can be seen that the controllers determine different values for the control variables. Therefore, for this trajectory, the occurring robot configurations are also different depending on the controller. Furthermore, it is noticeable that the calculated values for the control variables are larger than in the case of the swing motion. For the kinematic controller, the maximum value of the control, 43.85 N, is more than twice as large as the 20 N considered during the design.

## 5. Robustness against Parameter Uncertainty

Finally, the robustness of the different control concepts against deviations in the material parameters of the robot to be controlled is investigated. Especially for soft materials, the material parameters are often not known precisely, contain unmodeled nonlinear effects, are hard to determine and might even change over time [47]. Therefore, a robustness of the controllers against parameter uncertainty is of importance.

In order to examine the influences that parameter uncertainties have on the control performance of the different controllers, deviations in the value of the Young’s modulus *E* are considered as examples. It is often difficult to determine this value exactly, and at the same time, it has a large influence on the static and dynamic properties of the soft robot.

For the examination, for each controller, five simulations with different values of the Young’s modulus in the simulation are performed. Thereby, the nominal value of the Young’s modulus $E_{0}=7.32\;\times \;10^{5}\;Pa$ as well as a change in the Young’s modulus by ±25% and ±50% are considered. In all cases, the controllers are designed for the nominal Young’s modulus $E_{0}$. Note that a change in the Youngs’s modulus also affects the internal damping of the soft robot model since, in this work, stiffness-proportional damping is assumed. As an example, in this work, only the tracking of the swing trajectory is considered in the robustness analysis. For the other trajectories, qualitatively comparable results could be achieved.

**Figure 16:**
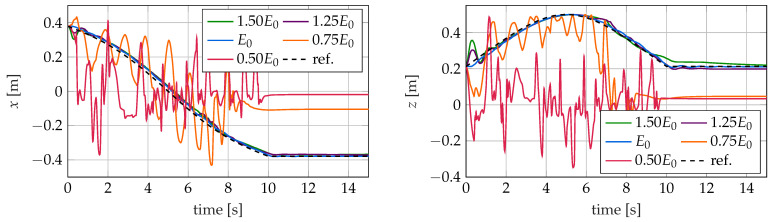
Trajectories of the tip position for the kinematic controller for different values of the young’s modulus *E*.

**Figure 17:**
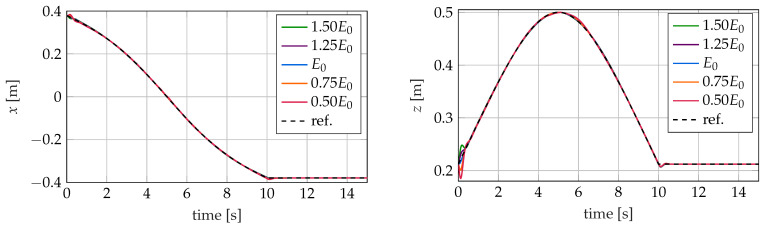
Trajectories of the tip position for the LQRI for different values of the young’s modulus *E*.

**Figure 18:**
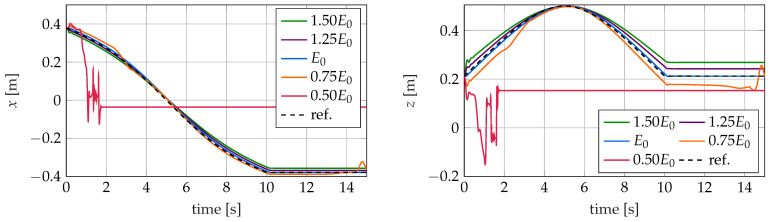
Trajectories of the tip position for the MPC for different values of the young’s modulus *E*.

In Figure 16, Figure 17 and Figure 18, the trajectory of the tip position is plotted for all three controllers and different values of the Young’s modulus in the simulation model. As described in Section 5, the controllers are designed for the nominal modulus of elasticity $E_{0}$. The different controllers react very differently to this modeling error.

### 5.1. Kinematic Controller

The kinematic controller again shows the worst performance of the three controllers. While the control of the robot with increased Young’s moduli ($E>E_{0}$) shows similar control results as those obtained when considering the nominal system with $E=E_{0}$, the determined tip position for softer robots with $E<E_{0}$ differs significantly. For these, an unsteady oscillatory behavior is shown before the tip comes to rest after the reference position no longer changes. However, the rest position is far away from the desired position. The reason for this is that due to the lower Young’s modulus, the applied control forces result in significantly larger motions than expected by the controller. As a result, the tip of the soft robot clearly leaves the workspace considered when designing the controller. Additionally, these large motions strongly excite the dynamics of the soft robot, which only decay very slowly. This makes the control for the kinematic controller even more difficult. Thus, the controller is no longer able to determine the required control variables with sufficient accuracy. This leads to the observed unsteady oscillation behavior. This behavior is also favored by the change in the dynamic behavior of the soft robot and the decrease in its internal damping. If, on the other hand, the Young’s modulus of the robot is greater than assumed by the controller, control variables that are too small are initially applied. In this case, the robot remains in its workspace, the dynamics of the controller are only slightly excited and the controller achieves a control result that is almost as good as that with the nominal Young’s modulus. The higher internal damping also probably has a positive influence on the achieved control quality.

### 5.2. LQI Controller

In contrast to the other two controllers investigated, the LQI controller shows no noteworthy differences between the trajectory tracking with the changed Young’s modulus and the nominal Young’s modulus of the simulation. Note that the gains of the controller are only determined for the nominal values. There are only minor deviations at the beginning of the movement. The reason for this is the changed static behavior of the soft robot model. As a result, the controller initially determines the control variables that belong to the equilibrium position of the robot at the current operating point with a comparatively large error. This is compensated by the controller after a short time. The LQI controller has no difficulties with the changes also occurring in the dynamic behavior of the controlled robot. The reasons for this are the integral component of the controller, the large gain of the controller and the high sampling frequency of f = 1 kHz, which allows the controller to react quickly to changes.

### 5.3. MPC

Furthermore, for the model predictive controller, differences in the achieved control performance and occurring deviations can be observed depending on the Young’s modulus of the controlled system. For stiffer robots with $E>E_{0}$, the *z* component of the tip position is permanently above the corresponding reference curve. The curvatures of the controlled robot turn out to be too small. As in the case of the kinematic and the LQI controller, this is due to the changed static properties of the soft robot. The control variables of the model predictive controller are determined by solving an optimization problem in such a way that they lead to the desired motion in the controller-internal model that uses the nominal parameters. If these are applied to the system to be controlled with a larger modulus of elasticity, there is less curvature than predicted by the controller due to the stiffer behavior of the robot. The too-low curvature for stiffer robots with $E>E_{0}$ can also be seen in the course of the *x* component of the tip point trajectory.

The presented effects also occur, with the same reasoning but opposite sign, for the softer robot with $E=0.75E_{0}$. For this robot, the curvature is smaller than predicted by the controller. If the elastic modulus of the controlled robot is further reduced, see $E=0.5E_{0}$, it becomes apparent that the MPC is no longer able to follow the reference trajectory. The low stiffness, as well as the resulting change in dynamic and static behavior, causes the closed loop to become unstable. Thus, the model predictive controller calculates ever increasing control variables to follow the reference trajectory with the internal model. These lead to the fact that the curvature of the soft robot increases more and more, and finally, the soft robot just “rolls up”. From the time t = 1.3 s on, the optimizer does not converge anymore and is no longer able to determine a solution for the optimization problem on which the control is based. From this moment on, the control variables of the controller are kept constant at the last “successfully” determined value. Due to the internal damping, the soft robot oscillates to the corresponding equilibrium position and remains in this position.

## 6. Conclusions

In this article, one kinematic and two different dynamic control approaches were analyzed for soft robots. For this purpose, these controllers were used to track various trajectories in simulation using an exemplary soft robot model. All three control methods are suitable for the control of soft robots. However, they strongly differ in implementation effort, achievable accuracy and robustness. Therefore, for these controllers, different applications arise. The advantages and disadvantages of the controllers are explained in the following and summarized in Table 6.

### 6.1. Kinematic Controller

The simplest of the three controllers studied is the kinematic controller. This can be seen as the standard approach in soft robotics. This controller is model-free. The data required for training the neural network on which the controller is based can for example be determined experimentally. However, compared to the dynamic controllers, the archived control errors are large. This is especially the case for fast motions. Here, large oscillations around the desired trajectory often occur. Additionally, the examination of the robustness has shown that the stiffness of the robot must not be overestimated by the controller to allow trajectory tracking. Additionally, this controller requires very low computational effort. Therefore, the kinematic controller should be used if low online implementation effort is important and/or no accurate model of the soft robot is available, while the required movements of the robot are slow and medium control errors, as well as oscillations, can be accepted. This holds for most of the current soft robotic applications.

### 6.2. LQRI

In this paper, an LQI controller with gain scheduling was proposed, which has not been used before in soft robots’ literature. This is the most accurate and robust of the three examined controllers. At the differentiable parts of the trajectory, the control error is negligible. Small control errors can only be observed at the non-differentiable parts of the trajectory. The LQRI is very robust against parameter uncertainties. Even a large change in the Young’s modulus of ±50% leads to small control errors. The controller has moderate computational effort (11.2 μs per iteration on a standard PC). The LQRI is a good option if the performance of the kinematic controller is not good enough. It can archive much higher accuracy and robustness than the kinematic controller, resulting in much higher achievable speeds. However, the implementation and computational effort is higher.

### 6.3. MPC

The model predictive controller achieves a comparatively high accuracy as the LQRI. However, due to the implementation used in this contribution, a small time delay cannot be avoided. The controller is model-based and therefore requires an accurate model of the robot. The MPC is robust against moderate parameter uncertainties. However, the control error increases significantly with increasing difference between the controlled robot and the internal model of the robot. If the stiffness of the robot is strongly underestimated by the MPC, it fails to find a solution for the optimal control problem. The computational cost of the MPC is very high compared to the other two examined controllers, but depending on the choice of control and prediction horizon, it is real-time capable on a standard PC. The MPC is a good alternative to the LQRI, especially if an accurate model of the robot is available and high computational costs are not a problem. As an advantage, additional constraints, such as actuator limits and avoiding obstacles, can easily be included in the optimization problem.

### 6.4. Limitations and Perspectives

The main limitation of this work is that the results were only obtained in simulations so far. In the future, it is planned to experimentally investigate what extent the results can be transferred to applications with physical robots. Additionally, the controllers can be extended to the control of movements in 3D.

## Figures and Tables

**Table 1:** Comparison of state-of-the-art control approaches for soft robots.

Class	Study	Model	Control Approach	Type
kinematic, model-based	[7]	PCC	direct inversion of kinematics	open-loop
	[8,9]	PCC	differential inversion of kinematics	open-loop
	[10]	Cosserat rod	differential inversion of kinematics	open-loop
	[11]	PCC	inversion of kinematics by optimization	open-loop
	[12]	PCC	inversion of kinematics with Jacobian transpose approach	open-loop
kinematic, model-free	[13]	-	learning of inverse kinematics with NN; learning on model	open-loop
	[14]	-	learning of inverse kinematics with NN; learning on physical robot	open-loop
	[4,5,15]	-	learning of inverse kinematics with NN	closed-loop
	[16]	-	learning of inverse kinematics with multitask Gaussian Process (open-loop) + locally weighted projection regression (closed-loop)	closed-loop
	[17,18]	-	online learning of kinematic Jacobian by incrementally moving each actuator	open-loop
dynamic, model-based	[19]	PCC	sliding mode	closed-loop
	[20,21,22,23]	PCC	PD	closed-loop
	[24]	rigid multibody system	MPC	closed-loop
	[25]	linear model + online Jacobian update	MPC	closed-loop
	[26]	PCC	adaptive control	closed-loop
	[27]	port-Hamiltonian	energy-shaping	closed-loop
dynamic, model-free	[28,29,30]	-	reinforcement-learning with Markov decision process	closed-loop
	[31]	-	supervised learning with NN	closed-loop
	[32,33,34]	-	MPC with NN model of soft robot	closed-loop

**Table 2:** Parameters of the simulation model.

**Variable**	**Description**	**Value**
$L_{\mathrm{total}}$	total length	500 mm
*r*	inner radius	25 mm
*R*	outer radius	50 mm
$r_{\mathrm{cable}}$	distance of cables to centerline	25 mm
*E*	Young’s modulus	$7.32\;\times \;10^{5}$ Pa
Eμ	damping constant	$36.6\;\times \;10^{3}$ Pas

**Table 3:** Characteristic quantities of the step trajectory with $d_{\mathrm{step}}=50\;mm$.

	Kinematic	LQI	MPC
rise time (10%…90%)	237.3 ms	90.2 ms	86.2 ms
settling time (2% band)	7.65 s	0.88 s	0.49 s
overshoot	47.46%	46.36%	31.49%
steady-state error	4.85%	<0.01%	<0.01%

**Table 4:** Characteristic quantities of the step trajectory with $d_{\mathrm{step}}=\;200\;mm$.

	Kinematic	LQI	MPC
rise time (10%…90%)	248.9 ms	78.6 ms	66.6 ms
settling time (2% band)	14.10 s	1.61 s	1.37 s
overshoot	53.71%	54.84%	20.51%
steady-state error	2.10%	<0.01%	<0.01%

**Table 5:** Computation time and sample time of the three controllers for the step trajectory.

Controller	Sample Time	Calculation Time per Control Step
kinematic	200 ms	5.4 μs
LQRI	1 ms	11.2 μs
MPC	100 ms	49.7 ms

**Table 6:** Advantages and disadvantages of the examined controllers.

	Kinematic	LQRI	MPC
Online/offline implementation effort	low	medium	high
Computation effort	very low	medium	high
Accuracy	low	very high	high
Robustness	low	very high	high

## Data Availability

Not applicable.

## Abbreviations

The following abbreviations are used in this manuscript:

LQI	Linear quadratic (regulator) with integral action
LQR	Linear quadratic regulator
LQRI	Linear quadratic regulator with integral action
LWPR	locally weighted projection regression
MPC	Model predictive controller
PCC	Piecewise constant curvature
PD	Proportional derivative [controller]
